# 定量检测BCR-ABL(P210)转录本水平室内质控体系的完善及多中心推广应用

**DOI:** 10.3760/cma.j.issn.0253-2727.2022.07.006

**Published:** 2022-07

**Authors:** 娜 何, 朝阳 谷, 乾鹏 李, 辉 周, 丽娜 初, 道新 马

**Affiliations:** 1 山东大学齐鲁医院血液病研究室，济南 250012 Institute of Hematology, Qilu Hospital of Shandong University, Jinan 250012, China; 2 潍坊市人民医院血液科，潍坊 261000 Department of Hematology, Weifang People's Hospital, Weifang 261000, China; 3 济宁医学院附属医院血液科，济宁 272007 Department of Hematology, Affiliated Hospital of Jining Medical University, Jining 272007, China; 4 烟台毓璜顶医院检验科，烟台 264099 Clinical Lab, Yantai Yuhuangding Hospital, Yantai 264099, China

**Keywords:** 聚合酶链反应, 融合蛋白质类，BCR-ABL, 室内质量控制, 推广应用, Real-time polymerase chain reaction, Fusion proteins, BCR-ABL, Internal quality control, Popularization and application

## Abstract

**目的:**

建立完善的BCR-ABL（P210）监测室内质控体系，确保检测结果的长期稳定性和室间可比性，并在3家医院进行推广应用。

**方法:**

山东大学齐鲁医院血液病研究室（H1）利用实时定量PCR（RQ-PCR）检测质控物的BCR-ABL（P210）转录本水平，完善质控体系的可靠性和稳定性；赴其他3家医院（H2～H4）现场进行测定前质量控制检查，同时邮寄给各家医院25套质控物进行检测；采用Levey-Jennings质控图结合Westgard多规则方法，对各家医院的标准曲线斜率、截距及检测结果进行统计学的质量判断，作出质控评价。

**结果:**

①成功建立并完善了测定前质控检查、测定中质控统计判断、测定后质控评价的BCR-ABL（P210）转录本水平监测的室内质控体系。②4家医院标准曲线的斜率、截距均在控。③多中心质控物判断结果：H1医院中、低浓度质控物各出现1次“1_2s_”警告，判断为在控；H2医院3种浓度质控物各出现1次“1_2s_”警告，判断为“2_2s_”失控；H3医院高浓度质控物违反1次“1_3s_”规则，低浓度质控物出现1次“1_2s_”警告，判断为“1_3s_”失控；H4医院各质控物均在控。④质控评价及纠正：2家医院在控，另2家医院各出现1次失控，查找原因进行纠正后未再出现失控。⑤多中心质控物原始数值比较：4家医院之间高浓度质控物结果差异有统计学意义，中、低浓度质控物结果差异无统计学意义。⑥多中心质控物国际标准值（IS值）比较：H2、H3医院的IS数值显著高于H1医院，H2医院显著高于H3医院。

**结论:**

建立了一套完善稳定的BCR-ABL（P210）转录本室内质控体系，有效地保证临床检测结果的稳定性和室间可比性，并成功完成了多中心的推广应用。

## Abstract (en)

**Objective:**

The study aims to establish a perfect BCR-ABL（P210）internal quality control system and ensure the long-term stability and comparability of the detection results between laboratories and to popularize and apply it in the three hospitals.

**Methods:**

The Qilu Hospital of Shandong University (H1) prepared a set of the BCR-ABL (P210) quality control substances to establish and improve internal quality control system. We went to other three participating hospitals (H2, H3, and H4) to inspect quality control before the measurement. In addition, we mailed 25 sets of quality control substances to each of the hospital for detection. The slope and intercept of the standard curve of each hospital and the detection results were analyzed and statistically judged using the Levey-Jennings quality control chart combined with the Westgard multirule theory. Then, we made a quality control evaluation.

**Results:**

①An internal quality control system for the BCR-ABL (P210) transcript levels monitoring was successfully established for the quality inspection before the measurement, statistical judgment during the measurement, and evaluation after the measurement. ② Both the slope and intercept of the standard curve of the four hospitals was under control. ③The multicenter quality control substance judgment results were as follows: for H1 hospital, two times of “1_2s_” warning were found in the middle-level quality control substance, which was judged as being under control; for H2 hospital, one time of “1_2s_” warning was found for each quality control substance, which was judged as being “2_2s_” out of control; for H3 hospital, its high-level quality control substance violated the “1_3s_” rule, and low-level quality control substance appeared “1_2s_” warning, which was judged as “1_3s_” out of control; and all quality control substances were under control in H4 hospital. ④The quality control evaluation and correction were as follows: two hospitals were under control, and the other two hospitals had an “out of control.” We found out the reason for the out of control and corrected them. ⑤The comparisons of the original values of the multicenter quality control substance were as follows: there were statistical differences in the results of high-level quality control substance among the four hospitals, and no significant difference was found in the results of the medium-level and low-level quality control substance. ⑥The comparisons of the IS values of the multicenter quality control substance were as follows: the IS values of the three quality control substance in H2 and H3 hospitals were significantly higher than those of H1 hospital, and H2 hospital was significantly higher than H3 hospital.

**Conclusion:**

A perfect and stable internal quality control system for the BCR-ABL (P210) transcripts has been established, which can effectively ensure the accuracy and stability of the clinical detection results. This internal quality control system has been successfully popularized and applied in other hospitals.

BCR-ABL（P210）融合基因是慢性髓性白血病（CML）最重要的分子学标志，更是评估CML患者治疗效果的决定性指标。实时定量PCR（RQ-PCR）检测BCR-ABL（P210）转录本水平对CML患者的病情判断、疗效监测及停药试验至关重要[1]。因此，CML患者在使用酪氨酸激酶抑制剂（TKI）治疗时，确保BCR-ABL（P210）转录本水平检测结果的准确性、长期稳定性和实验室间的可比性显得尤为重要。室内质量控制（internal quality control, IQC）作为全面质量管理体系的重要环节，是检验实验室检测结果准确性、稳定性并及时发现检测过程中实验偏差的管理体系，其主要包括三个方面：测定前的质量控制检查、测定中的统计学质量控制判断和测定后的质量控制评价[2]。血液病实验室实行IQC旨在对血液病检测数据的真实性、准确性、有效性进行评价，保证血液病检验工作的可靠性。因此，本研究在前期已初步建立的质控物检测方法的基础上[3]，将完善的IQC体系引入BCR-ABL（P210）检测中，在做好测定前质量控制检查的基础上，采用自制的新质控物连续监测BCR-ABL（P210）转录本水平，绘制质控图，结合Westgard多规则方法[4]–[7]，进行统计学质量控制判断，最后对本实验室检测结果进行质控评价，建立完善的BCR-ABL（P210）监测IQC体系，并将此质控体系在其他3家医院进行推广应用。

## 材料与方法

一、仪器与试剂

实时荧光定量PCR仪（ABI 7500）和CO_2_培养箱均购自美国Thermo Fisher公司，常温离心机购自北京白洋医疗器械有限公司，生物安全柜购自上海力申科学仪器有限公司，低温冰柜购自松下（大连）有限公司；BCR-ABL（P210）转录本检测试剂盒购自上海源奇医药科技有限公司，RPMI 1640培养基、TRIzol试剂购自美国Thermo fisher公司，胎牛血清购自以色列Biological Industries公司。

二、BCR-ABL（P210）转录本室内质控体系的建立及完善

1. 测定前的质量控制检查：

（1）实验人员培训及标准操作程序（SOP）制定：对实验室工作人员进行质控重要性、实验理论和技术的培训，熟练BCR-ABL（P210）检测的操作技能。严格制定一套完整的SOP，保证技术操作的规范性。

（2）仪器维护及试剂确定：对定量PCR仪、移液器、离心机、分光光度计等设备每半年校准1次；购买3个批号的BCR-ABL（P210）试剂盒，使用旧质控物测试[3]，选择检测结果符合质控体系的批号试剂，进行一年用量的购买，保证检测结果的稳定性。

2. 测定中的统计学质量控制判断：

（1）质控物的制备：在前期已有质控物检测的基础上[3]，收取本实验室培养的K562细胞［BCR-ABL（P210）阳性］，加入Trizol充分裂解，使每管1 ml Trizol中含5×10^5^细胞，作为高浓度质控物；同样收取Jurkat细胞系［BCR-ABL（P210）阴性］，将K562细胞与Jurkat细胞分别按1∶500、1∶1500稀释，使每管1 ml Trizol中含5×10^5^细胞，分别作为中、低浓度质控物，其主要侧重主要分子学反应（MMR）低水平的检测。上述质控物进行RQ-PCR检测，得到的结果乘以本院转换系数（CF）0.36，获得国际标准值（IS）。将上述3种质控物分别分装到500个1.5 ml Ep管中，进行编号，分为500套质控物，每套含高、中、低浓度质控物各1管，保存于−80 °C冰柜中。

（2）质控物Levey-Jennings（L-J）质控图的绘制[4]–[5]：常规工作中，每次将1套质控物的3管与患者样本同样条件下检测，记录各质控物检测结果，输入Excel表格，计算均值（*x*），作为中心线，以*x*±1s为控制线，*x*±2s为警告线，*x*±3s为失控线，绘制L-J质控图。

（3）Westgard多规则质控判断[6]–[7]：对生成的L-J质控图，利用Westgard多规则标准进行判断，质控规则主要为：①1_3s_失控规则：3份质控物中的任意1份测定值超过*x*±3s失控线，判定为“失控”；②1_2s_警告规则：3份质控物中的任意1份测定值处于*x*±2s～*x*±3s警告线，判定为“警告”。③2_2s_失控规则：同批两个质控物同方向超出*x*±2s警告线，或同一质控物连续两次超出*x*±2s警告线，判定为“失控”；④R_4s_失控规则：同批两个质控物之差超出4s范围，其中一个超出*x*+2s警告线，另一个超出*x*−2s警告线，判定为“失控”；⑤4_1s_失控规则：1份质控物结果连续4次同方向超过*x*±1s控制线，或2份质控物结果同时连续2次超过*x*±1s控制线，判定为“失控”；⑥10_*x*_规则：1份质控物结果连续10次偏于一侧时，或2份质控物结果同时连续5次偏于均值一侧时，判定为“失控”。

将不同水平的质控测定值转换成*Z*分数形式，绘制*Z*−分数质控图。根据Westgard多规则控制方法，当*Z*>2或*Z*<−2时，即违反1_2s_警告规则；当*Z*>3或*Z*<−3时，即违反1_3s_规则，为失控。

3. 测定后的质量控制评价：利用上述规则对结果进行判断，分析每一批次是否在控。对失控批次查找原因，采取措施进行纠正，从而验证质控体系的稳定可信性。

三、BCR-ABL（P210）转录本检测室内质控体系的多中心推广应用

1. 测定前的质量控制检查：为验证BCR-ABL（P210）室内质控体系的实用性，本院（H1）联合其他3家医院（H2～4）进行该室内质控体系的推广应用。测定前分别在4家医院现场进行仪器、试剂、人员、SOP、污染控制等各方面的标准化培训。H1、H2和H3医院均使用ABI 7500定量PCR仪，H4医院使用LC480 Ⅱ定量PCR仪。试剂均购自上海睿昂基因科技有限公司。

2. 室内质控物的派发与检测：我院检测25套质控物（每套含高、中、低浓度各1管），同时干冰保存下分别快递至其他3家医院各25套质控物，4家医院根据自己实际情况进行RNA提取、逆转录及BCR-ABL（P210）转录本的检测。为真实反映各个实验室的水平，建议每家医院将25套样本在3个月内分散检测。

3. 统计学质量控制及评价：BCR-ABL（P210）转录本检测时的标准曲线，与质控物的检测结果密切相关，监测斜率和截距的变化可以反映试剂的质量和PCR的扩增效率[8]。对4家医院BCR-ABL（P210）转录本定量PCR检测的标准曲线斜率、截距及质控物检测结果，分别用Graphpad 6.02统计软件进行方差齐性检验和方差分析，以*P*<0.05为差异有统计学意义。同时，用Excel绘制质控物结果的L-J质控图，并对质控数据采用1_3s_/2_2s_/R_4s_/10_*x*_的Westgard多规则质控方法进行评价[3]，对失控情况查找原因并进行纠正。

## 结果

1. 多中心检测标准曲线的结果分析：本研究在认定4家医院测定前质量控制均合格的基础上，统计分析其标准曲线的斜率、截距，结果如表1所示；同时对斜率、截距绘制Z分数质控图并进行Westergard多规则判断，结果显示4家医院的斜率、截距均在控（图1），说明检测结果可信，PCR扩增体系稳定。其中，H2斜率、截距的变异系数（CV值）最小，表明其检测结果的波动性小，较其他3家医院更加稳定。

**表1:** 4家医院BCR-ABL（P210）检测标准曲线斜率、截距

医院	检测次数	斜率			截距		
		*x*	*s*	CV	*x*	*s*	CV
H1	25	−3.45	0.20	5.88	40.97	0.98	2.40
H2	25	−3.36	0.10	3.10	40.45	0.93	2.29
H3	25	−3.37	0.18	5.32	42.13	1.13	2.67
H4	25	−3.31	0.18	5.49	39.89	1.02	2.55

注：CV：变异系数

**图1:**
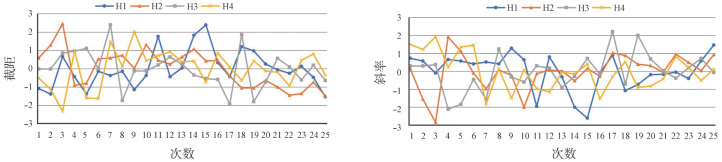
4家医院BCR-ABL（P210）检测标准曲线斜率、截距Z分数质控图

2. 室内质控体系的成功建立及完善：为验证本质控体系的可靠性、稳定性，我们在分装的500套质控物中随机抽取20套进行检测，绘制L-J质控图，利用Westgard规则进行判断，发现三种质控物均在控（图2）。我们也用同样方法，在质控物分装后的第3、6、9、12个月时，每次连续监测高、中、低浓度质控物3次，发现结果均在控（图3）。表明本质控物在管间一致性、时间稳定性方面都是可信的。

**图2:**
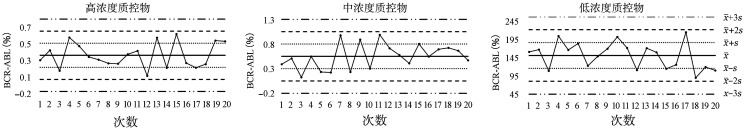
随机抽取20套的3种浓度质控物L-J质控图

**图3:**
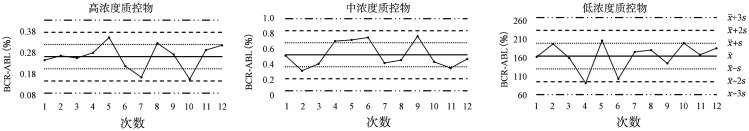
不同时间点检测3种浓度质控物L-J质控图

在此基础上，本院继而在3个月内连续进行另外20次检测，绘制L-J质控图（图4），根据Westgard质控规则判断发现，3种质控物各有1次1_2s_警告（高浓度质控第10次、中浓度质控第2次、低浓度质控第7次），未出现失控情况；同时CV值分别为33.32、35.67、34.16。说明本院新建立的BCR-ABL（P210）转录本室内质控体系稳定可信。

**图4:**
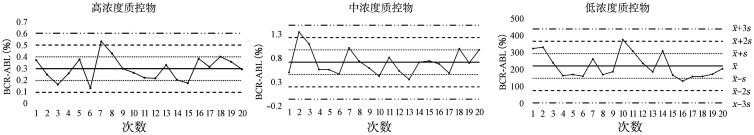
本院3种浓度质控物结果L-J质控图

3. 多中心室内质控物的统计学质控判断结果：四家医院分别以各自3种质控物的25次检测结果绘制L-J质控图，然后应用Westgard多规则方法进行判断分析。结果显示，H1医院中浓度质控物第20次和低浓度质控物第24次均出现“1_2s_”警告，判断为在控；H2医院高浓度质控物第8次、中浓度质控物第9次和低浓度质控物第8次均出现“1_2s_”警告，判断为“2_2s_”失控；H3医院高浓度质控物第24次违反“1_3s_”规则，低浓度质控物第22次出现“1_2s_”警告，判断为“1_3s_”失控；H4医院的3种质控物均在控。

4. 室内质控的评价及偏差纠正：对质控结果进行评价，2家医院（H1、H4）质控结果均在控，另2家医院（H2、H3）质控结果各存在1次失控。通过及时查找原因，发现H2医院由于定量PCR仪的温控板出现故障导致受热不均匀，造成质控结果出现了较大偏差，仪器经过更换部件，重新校准后纠正了失控结果；H3医院由于更换试剂批号造成检测结果的波动，通过试剂调整后纠正了偏差。

5. 室内质控物原始检测数值与IS值的多中心比较：如图5所示，对4家医院质控物的原始检测数值进行比较，发现高浓度质控物结果差异有统计学意义（*P*<0.001），中、低浓度质控物结果差异均无统计学意义（*P*值均>0.05）。将具有CF的3家医院（H1、H2和H3）的原始检测值乘以自身CF，转换成IS值后进行统计分析，发现H2、H3医院3种质控物的IS数值均显著高于H1医院，且H2医院高于H3医院，差异均具有统计学意义（*P*值均<0.05）。

**图5:**
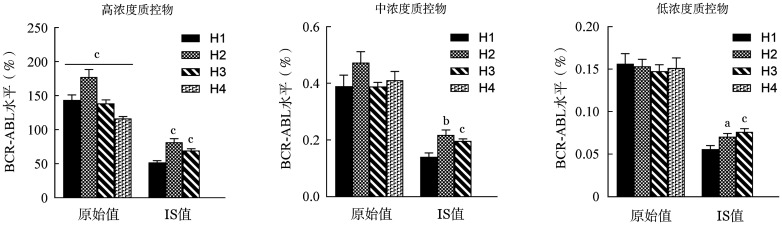
多中心质控物检测结果的原始值及国际标准值（IS）值比较（与H1医院比较，^a^*P*<0.05，^b^*P*<0.01，^c^*P*<0.001）

## 讨论

IQC是血液病实验室内部最基本的质量监督、检测、评价和改进体系，是全面质量管理体系中的一个重要环节[4]。虽然澳大利亚Adelaide实验室等在此方面做了一些工作[9]–[12]，但目前尚无统一的BCR-ABL（P210）室内质控标准。因此，本研究结合日常工作实际，在前期工作[3]以及做好测定前质量控制的基础上，对所建立的BCR-ABL（P210）的IQC监测方法进行完善。首先，本体系中扩增反应的标准曲线斜率、截距结果均在控，表明试剂是稳定的，如果一旦同批号试剂标准曲线斜率与截距的CV值过大，应即刻查找原因并采取对策。然后，随机抽取分装后的3种浓度的质控物进行检测，同时也在保存的不同时间点进行检测，结果显示均在控，从而有效保证了本质控体系在不同管间的可比性和质控物长期保存方法的稳定性。我们也利用质控物在3个月内进行20次检测，发现3种质控物均未出现失控情况。变异系数CV是标准差与平均值之比用百分数表示，Adelaide实验室2001–2007年的质控数据显示CV值越小，数据波动性越小，表明质控结果越稳定[10]。我们与澳大利亚Adelaide实验室的CV值非常相似，说明本院新建立的BCR-ABL（P210）转录本室内质控体系稳定可信。

由于RQ-PCR的多步骤性，世界各地实验室使用的方法和程序各不相同，使用不同的标本、不同的RNA制备方法、不同的逆转录和PCR试剂、不同的标准品、不同的内参基因监测RNA的数量和质量以及不同的仪器等，这些因素都导致了BCR-ABL（P210）转录本的变化[13]。亦有文献报道，在实际的临床工作中，CML患者单独一次的BCR-ABL（P210）检测结果对临床的指导意义并不是很大，应该观察BCR-ABL（P210）转录本水平的连续变动趋势；同时由于PCR技术中多种影响因素带来的固有误差，完全相同的两个样本检测结果可能存在较大的差异，如果实际临床工作中CML患者连续两次检测结果差异≤1个数量级（或者≤10倍），可以认为是系统误差造成的，不能视其为BCR-ABL（P210）的真正生物学变化[14]，应该在室内质控可靠的基础上重新抽取样本进行复测以核实结果的可信性。

任何一个Westgard判断规则的失控，都可能造成患者检测结果的不正确，因此我们要及时、全方位去查找原因，争取在最短的时间内获得正确的结果。在本研究中，4家医院同时检测同一批质控物，我们通过对质控结果进行质控评价，发现H2、H3质控结果各存在1次失控。及时查找失控原因，发现H2医院由于定量PCR仪温控板出现故障导致受热不均匀，造成质控结果出现较大偏差，仪器经过更换部件，重新校准后纠正了失控结果；H3医院由于更换试剂批号造成检测结果的波动，通过试剂调整后纠正了偏差。在前期研究中，我们发现更换试剂或质控物批号时，应以新的质控物或试剂重新检测至少20次，累积数据绘制新质控图或校正转化后使用原质控图，并且我们根据自身实验室情况探索出质控图的延续方法，通过对新旧批次的试剂或质控物数据进行比较和差异分析，再通过均数的倍数转换后使用原质控图，质控结果满意[3]。在本研究中，4家医院均存在检测过程中更换试剂批号的现象。试剂更换可能会影响BCR-ABL（P210）转录本水平的检测值及CF值，因此不建议频繁更换试剂批号，更不要频繁更换生产厂家。若要保证获得的CF值持续有效，自身实验室须严格执行标准化操作方案，严格进行室内质控，一旦检测方案有变化，需重新验证CF值。

4家医院质控物的原始检测数值中，高质控物结果差异有统计学意义，中、低质控物结果均未发现明显差异。而将具有CF的3家医院（H1、H2和H3）的检测值转换成IS值后，差异均具有统计学意义，表明3家医院检测结果原始检测数值的一致性均优于转换后的IS值，说明其中某个（些）医院以前获取的CF已不适用现在的检测结果，3家医院现在的CF理论上应该相同或极为相近。实际应用中，本室内质控体系不仅可以发挥IQC的作用，同时还可以作为室间质量评价（EQA）的质控物发挥作用。

目前，EQA的权威性高于IQC，但其成本高、对实验室样本数量要求比较高，可操作性一般。此外，EQA也是建立在IQC切实有效实施的基础上，因而IQC是实验室全部质量控制的基础。IQC开展的目的是保证检测过程精密、准确和可重复，使实验结果误差处于规定的置信区间内[15]。IQC主要措施是对同一样本重复多次进行测定，对测得结果的精密度和准确度进行评价和控制[16]。本研究选用相当于CML患者初诊、MMR水平以及更低水平的3个指标考核精密度，提高了临床检验室BCR-ABL（P210）监测的准确性，并且同时可以兼具IQC和EQA的性能，在多中心成功进行了初步的推广应用。

## References

[1] Patel S, Mistry P, Patel K (2020). Clinical Significance of BCR-ABL Fusion Gene in Chronic Myeloid Leukemia Patients[J]. J Assoc Genet Technol.

[2] 王 治国, 李 小鹏, 武 平原 (2004). 临床检验定量测定室内质控系统的建立[J]. 检验医学.

[3] 钟 朝琴, 何 娜, 华 明强 (2016). 实时定量PCR检测BCR-ABL (P210)转录本水平室内长期质控体系的建立及应用[J]. 中华血液学杂志.

[4] 王 惠民 (2012). 临床实验室管理学[M].

[5] 杨 勇, 毅 陈斌, 韩 军民 (2003). Levery-Jennings室内质控图的建立[J]. 临床输血与检验.

[6] 刘 保廷, 雷 桂华 (2004). 应用Levey-Jennings质控图结合Westgard多规则进行临床化学室内质控[J]. 现代检验医学杂志.

[7] 李 锐, 杜 威, 祁 百胜 (2013). 正确理解和应用Westgard多规则质控规则[J]. 中外健康文摘.

[8] 黄 学忠, 杜 笑雅, 吴 祥 (2004). 利用标准曲线斜率与截距对HBV DNA荧光定量PCR实施质量监控[J]. 临床检验杂志.

[9] Braun TP, Eide CA, Druker BJ (2020). Response and Resistance to BCR-ABL1-Targeted Therapies[J]. Cancer Cell.

[10] Branford S, Fletcher L, Cross NC (2008). Desirable performance characteristics for BCR-ABL measurement on an international reporting scale to allow consistent interpretation of individual patient response and comparison of response rates between clinical trials[J]. Blood.

[11] Hughes T, Deininger M, Hochhaus A (2006). Monitoring CML patients responding to treatment with tyrosine kinase inhibitors: review and recommendations for harmonizing current methodology for detecting BCR-ABL transcripts and kinase domain mutations and for expressing results[J]. Blood.

[12] Stanoszek LM, Crawford EL, Blomquist TM (2013). Quality control methods for optimal BCR-ABL1 clinical testing in human whole blood samples[J]. J Mol Diagn.

[13] Zhang T, Grenier S, Nwachukwu B (2007). Inter-laboratory comparison of chronic myeloid leukemia minimal residual disease monitoring: summary and recommendations[J]. J Mol Diagn.

[14] Akard LP, Wang YL (2011). Translating trial-based molecular monitoring into clinical practice: importance of international standards and practical considerations for community practitioners[J]. Clin Lymphoma Myeloma Leuk.

[15] 李 润青, 宫 丽君, 王 腾蛟 (2017). 西格玛方法在临床生化检验质量管理中的应用[J]. 中华检验医学杂志.

[16] 朱 琳, 张 松, 郝 军 (2016). 白介素6上调糖尿病肾小管上皮细胞脂肪分化相关蛋白[J]. 中国组织化学与细胞化学杂志.

